# Live birth rate and neonatal outcomes following interventional embolization of hydrosalpinx

**DOI:** 10.1186/s12978-022-01522-7

**Published:** 2022-12-01

**Authors:** Haiyan Guo, Tong Du, Qifeng Lyu, Ling Wu, Weiran Chai, Qianqian Zhu

**Affiliations:** Department of Assisted Reproduction, Shanghai Ninth People’s Hospital, Shanghai Jiaotong University School of Medicine, Center for Specialty Strategy Research of Shanghai, Jiao Tong University China Hospital Development Institute, Shanghai, 200011 China

**Keywords:** Hydrosalpinx, Frozen-thawed embryo transfer, Reproductive outcome, Embolization

## Abstract

**Background:**

Hydrosalpinx has a negative effect on the pregnancy outcomes of in vitro fertilization and embryo transfer (IVF-ET), and the pretreatment for hydrosalpinx play an important role in improving the outcomes of IVF-ET. This study aimed to investigate the impacts of interventional embolization of hydrosalpinx on the live birth rate and neonatal outcome after in-vitro fertilization.

**Method:**

In the present retrospective study, 3351 women receiving the first frozen embryo transfer (FET) after freeze-all policy were reviewed. Patients who received interventional embolization of hydrosalpinx (n = 1268) were included in the study group and those with hydrosalpinx-free bilateral fallopian tube obstruction (n = 2083) in the control group. The primary outcome was live birth (LB) rate; the secondary endpoints included rates of implantation, clinical pregnancy (CP), multiple pregnancy, and pregnancy loss.

**Results:**

The LB rate was similar between embolization group (39.91%) and control group (43.21%) (P > 0.05). The rate of implantation (35.81% vs. 32.24%), CP (50.84% vs. 47%) and multiple pregnancy rate (28.71% vs. 24.16%) in the control group were significantly higher than in the embolization group (P < 0.05). The miscarriage rate (39.91%, vs 43.21%, P > 0.05), ectopic gestation rate (2.35% vs 2.83%, P > 0.05), and ongoing pregnancy rate (41.56% vs 44.89%, P > 0.05) were comparable between two groups. After adjustment for confounding factors, interventional embolization of hydrosalpinx was found to have no influence on the LB rate. The thicker endometrium, more embryos transferred, and transfer of blastocyst stage embryos significantly increased the LB rate and CP rate.

**Conclusion:**

The interventional embolization of hydrosalpinx can achieve the LB rate similar to that of hydrosalpinx-free obstruction patients with less risk, less pain and reduced medical cost. Thus, embolization of hydrosalpinx is one of the preferable clinical treatments for patients with hydrosalpinx.

## Plain English summary

Our study indicates that interventional embolization of hydrosalpinx is one of the preferred clinical treatments for hydrosalpinx. More studies are warranted to investigate the short- and long-term effects of interventional embolization on the outcomes of pregnant women and neonates, which may provide more information about the management of hydrosalpinx.

## Background

As a place for the fertilization of sperm and eggs and for the transport channel of fertilized eggs, the fallopian tubes play an important role in the reproductive process [1]. Any abnormality in the fallopian tubes may lead to infertility. According to the statistics, fallopian tube diseases are the main cause of infertility (accounting for 40%), while hydrosalpinx accounts for 10–30% of various fallopian tube diseases [2]. Since the introduction of in vitro fertilization and embryo transfer (IVF-ET), the infertility has been significantly improved.

However, the efficacy of IVF-ET is often unsatisfactory for patients with hydrosalpinx. It has been reported that hydrosalpinx is related to the reduced implantation rate and clinical pregnancy rate following IVF-ET, and associated with increased early abortion rate and ectopic pregnancy rate [3]. This might be ascribed to the mechanical erosion [4], toxic effect of embryo and gamete [1, 2], and reduced endometrial receptivity [3]. Therefore, pretreatment for hydrosalpinx is of great significance to improve the outcome of IVF-ET. Current treatments for hydrosalpinx mainly include ultrasound-guided hydrosalpinx aspiration, fimbria salpingostomy, proximal tubal ligation, and tubectomy [4, 5]. Hydrosalpinx aspiration and salpingostomy may cause high rates of hydrosalpinx recurrence and ectopic gestation [6]. Although tubectomy can significantly improve the pregnancy outcome of patients with hydrosalpinx, it may cause damage to the arteries of fallopian tube-mesovarium, which affects the ovarian blood flow and reduces ovarian reserve function and superovulation response [7]. Recently, with the development of interventional radiology, interventional embolization has been applied in the treatment of hydrosalpinx before IVF-ET, achieving favorable therapeutic efficacy. The interventional embolization has the advantages of convenience, low cost, no anesthetic risk, and no influence on the ovarian function, and can significantly improve the pregnancy rate and reduce ectopic gestation rate [8, 9]. The frozen-thawed embryo transfer (FET), which allows the transfer of embryos into a more physiological uterine environment, has attracted much attention in recent years [10]. Few studies have been designed to evaluate the impact of hydrosalpinx on the FET cycles. In a more recent study, 129 women who received tubal interventional embolization before IVF-ET were investigated, and results showed the interventional embolization for hydrosalpinx before IVF-ET could improve the clinical pregnancy rate and reduce the adverse pregnancy outcomes, but had no influence on the ovarian function [11].

Currently, the effects of different managements of hydrosalpinx on the rates of implantation, clinical pregnancy and live birth have not been thoroughly studied. Thus, the present study was to investigate the effects of interventional embolization of hydrosalpinx on the pregnancy outcomes in a large cohort of women undergoing first FET cycle in our center after controlling for ovarian factors (only patients with high-quality embryo transfer were included).

## Methods and materials

### Study design and population

This was a retrospective study conducted at the Department of Assisted Reproduction of the Ninth People’s Hospital of Shanghai Jiao Tong University School of Medicine. Women who underwent first FET cycle in our center and had high-quality embryo transfer (described below) following a freeze-all policy between January 2010 and June 2019 were included into present study. Patients who received the interventional embolization of hydrosalpinx (n = 1268) were included in the embolization group, and those with hydrosalpinx-free bilateral fallopian tube obstruction (obstruction group; n = 2083) in the control group. Exclusion criteria were as follows: (1) patients were older than 40 years; (2) patients had adenomyosis/endometrioma; (3) hydrosalpinx was untreated, or hydrosalpinx was treated by ligation or resection (4) patients had a history of recurrent miscarriage (≥ 2 previous biochemical/clinical losses); (5) patients had a prior IVF regardless of fresh or frozen embryo transfer; (6) patients had submucosal fibroids or polyps, intramural fibroids > 4 cm, or congenital uterine malformation on three-dimensional ultrasonography and hysterosalpingography; (7) patients had hypertension, diabetes, or thyroid dysfunction.

Patients were divided into two groups: 1268 patients with unilateral or bilateral hydrosalpinx in the embolization group were treated with interventional embolization of hydrosalpinx; in the same period, 2083 patients with hydrosalpinx-free bilateral fallopian tube obstruction were recruited as the control group. Interventional embolization had been introduced in previous studies [8], and could be completed in the outpatient operation room. Patients with normal parameters received interventional embolization after routine examinations of chlamydia, mycoplasma, and leucorrhea. Contralateral fallopian tube of patients with bilateral hydrosalpinx was examined with interventional embolization. Finally, hystero-salpingography (HSG) was employed to assess the efficacy of embolization. If hydrosalpinx was visible on ultrasonography on the day of transplantation, unilateral or bilateral hydrosalpinx was subjected to ultrasound-guided aspiration without uterine puncture on the day of FET if necessary.

This retrospective study was carried out in accordance with the Helsinki Declaration, and the study protocol was approved by the Ethics Committee (Institutional Review Board) of the Shanghai Ninth People's Hospital (SH9H-2021-T124-1). This study conforms with the Strengthening the Reporting of Observational Studies in Epidemiology (STROBE) Statement, validated by the Enhancing the Quality and Transparency Of health Research (EQUATOR) network.

### Endometrial preparation prior to embryo transfer

Modified natural cycles were applied for patients with regular ovulatory cycle. In patients with irregular menses, endometrial preparation was performed in either a mildly letrozole-stimulated cycle or an artificial cycle (AC), depending on patients’ preference and the discretion of clinicians. These methods used for endometrial preparation had been described in previous studies [12]. In both groups, luteal support was provided until 10 weeks of gestation if a pregnancy occurred.

### Embryo quality assessment and vitrification

Embryo morphology was evaluated on days 3, 5, and 6. Cleavage-stage embryos with at least 6 blastomeres and fragmentation < 20% were regarded as high-quality embryos [13]. Blastocysts were scored according to the Gardner and Schoolcraft grading system [14] and recorded as high-quality ones if they reached an expansion stage at least 4 with C or C for inner cell mass and trophectoderm (4CC). Of note, during the study period, the type of culture medium was completely changed. Specifically, commercially sequential available culture medium (Irvine Scientific) was used before January 2013. Continuous single culture media (Irvine Scientific) had been introduced since January 2013. The other laboratory procedures and conditions remained unchanged. The vitrification and thawing procedures were previously described by Kuwayama et al. [15]. Briefly, embryo vitrification was carried out via a Cyrotop carrier system, in conjunction with DMSO-EG-S as the cryoprotectant. For thawing, the embryos were transferred into dilution solution in a sequential manner (1–0.5–0 M sucrose).

### Outcomes

The primary outcome was the live birth rate. The secondary endpoints included the rates of implantation, clinical pregnancy, multiple pregnancy and miscarriage, and the perinatal outcomes. Live birth was defined as a live neonate born after 24 weeks of gestation. Ongoing pregnancy was defined as a viable pregnancy that progressed beyond 12 weeks of gestation. A clinical pregnancy was confirmed by the presence of a gestational sac on ultrasonography 5 weeks after embryo transfer. Miscarriage was defined as the loss of clinical pregnancy before the 24th week of gestation. The implantation rate was calculated as the number of gestational sacs visualized on ultrasonography divided by the number of embryos transferred. All calculations were made on a “per embryo transfer” basis. Low birth weight and macrosomia were defined as birth weight < 2500 g and ≥ 4000 g, respectively. Preterm birth was defined as a delivery before 37 weeks of gestation. Early premature term baby (PTB) was defined as the baby delivered before 32 weeks of gestation. The body weight between 1500 and 2500 g at birth was defined as low birth weight (LBW) and the body weight lower than 1500 g as very LBW. All neonatal and delivery information was obtained from the electronic medical records.

### Statistical analysis

The baseline characteristics and clinical outcomes were compared using t test or Chi-square test where appropriate. The multivariable logistic regression analysis was employed to determine the effects of different managements of hydrosalpinx on the live birth rate after adjustment for potential confounding factors, including maternal age, infertility duration, obstetric history (gravidity and parity), cause of infertility (tubal disease, endometriosis, male cause, diminish ovary reserve), number of ovum retrieved, failures of previous FET, number of embryos transferred, embryo stage at transfer, and type of cycle (IVF, intracytoplasmic sperm injection [ICSI] or IVF + ICSI). The women with tubal interventional embolization served as the embolization group. The adjusted odds ratios (aORs) and 95% confidence intervals (CIs) were calculated to show the strength of association. All statistical analyses were performed with the Statistical Package for Social Sciences (SPSS) version 21.0. A value of P < 0.05 was considered statistically significant.

## Results

A total of 3351 patients underwent first FET cycle during the study period. Baseline characteristics in different groups are shown in Table 1. Compared with the control group, the prevalence of primary infertility (50.95% vs. 58.09%, P < 0.05) and the number of previous FET in the embolization group were significantly lower in the embolization group. There were markedly higher gravidity and parity in the embolization group than in the control group. No significant differences were noted in the age (35.28 ± 2.91 vs. 34.90 ± 3.64, P > 0.05), body mass index (BMI), infertility duration, fertilization method, and number of oocytes retrieved between two groups.

**Table 1:** Characteristics of FET cycles in each group

Characteristics	Embolization group (n = 1268)	Obstruction group (n = 2083)	P
Maternal ages (year)	35.28 ± 2.91	34.90 ± 3.64	0.816
Maternal BMI (kg/m^2^)	21.50 ± 3.42	21.47 ± 2.85	0.097
Infertility duration (year)	3.20 ± 2.40	3.08 ± 2.37	0.067
Infertility type			
Primary infertility	646 (50.95)	1210 (58.09)	0.000
Gravidity			0.000
0	639 (63.17)	1207 (57.95)	
1	410 (32.33)	535 (25.68)	
≥ 2	219 (17.27)	341 (16.37)	
Parity			0.000
0	1199 (94.56)	1903 (91.35)	
1	69 (5.44)	165 (7.92)	
≥ 2	0 (0)	15 (0.72)	
Cause of infertility			0.000
Tubal factor	950 (74.92)	1552 (74.51)	
PCOS	59 (4.65)	115 (5.52)	
Endometriosis	84 (6.62)	92 (4.42)	
Male factor	148 (11.67)	311 (14.93)	
Diminish ovary reserve	27 (2.13)	13 (0.62)	
Number of previous FET failures			0.000
0	805 (63.49)	1500 (72.01)	
High order	463 (36.51)	593 (28.47)	
Fertilization method			0.75
IVF	984 (77.60)	1617 (77.63)	
ICSI	209 (16.48)	331 (16.37)	
IVF + ICSI	75 (5.91)	135 (6.48)	
Number of oocytes retrieved			0.349
1–10	586 (46.21)	913 (43.83)	
11–20	489 (38.56)	825 (40.09)	
> 20	193 (15.22)	345 (16.56)	

Data are presented as mean ± SD for continuous variables and n (%) for dichotomous variables

*P* Embolization group vs. Obstruction group; *FET* frozen-thawed embryo transfer; *ICSI* intracytoplasmic sperm injection; *IVF* in vitro fertilization; *PCOS* polycystic ovary syndrome

P < 0.05 indicated a significant statistical difference

The FET cycles in different groups are summarized in Table 2. There were no significant differences in the number of embryos transferred, embryo quality, protocol of endometrial preparation and endometrial thickness on the day of embryo transfer between two groups (P > 0.05). However, women in the embolization group more frequently experienced cleavage stage (day 3) in the embryo developmental stage at transfer (90.78% vs 82.29%, P < 0.01).

**Table 2:** Cycle characteristics of FET

Characteristics	Embolization group (n = 1268)	Obstruction group (n = 2083)	P
FET endometrial preparation			0.509
Natural cycle	463 (36.51)	777 (37.30)	
Artificial cycle	459 (36.20)	718 (34.47)	
LE stimulation cycle	346 (27.29)	598 (28.71)	
Number of embryos transferred			0.559
1	218 (11.19)	367 (17.62)	
2	1043 (82.26)	1698 (81.52)	
3	7 (0.55)	18 (0.86)	
Embryo developmental stage at transfer			0.000
Cleavage stage (day 3)	1151 (90.78)	1725 (82.29)	
Blastocyst (day 5/6)	117 (9.22)	358 (17.71)	
Endometrium thickness on the day of transfer (mm)	11.10 ± 2.37	11.05 ± 2.58	0.096
Post-thaw embryo survival rate	2340/2342 (99.91)	3837/3838 (99.97)	0.392

Data are presented as mean ± SD for continuous variables and n (%) for dichotomous variables

*P* Embolization group vs. Obstruction group; *FET* frozen-thawed embryo transfer

P < 0.05 indicated a significant statistical difference

The pregnancy outcomes are shown in Table 3. The LB rate was similar between embolization group (39.91%) and control group (43.21%, P > 0.05). The control group had higher rates of implantation (35.81% vs. 32.24%), CP (50.84% vs. 47%) and multiple pregnancy (28.71% vs. 24.16%) than the embolization group (P < 0.05). The miscarriage rate (39.91%), ectopic gestation rate (2.35%), and ongoing pregnancy rate (41.56%) in the embolization group were similar to those in the control group (43.21%, 2.83% and 44.89%, respectively, P > 0.05).

**Table 3:** Reproductive outcomes per embryo transfer

Characteristics	Embolization group (n = 1268)	Obstruction group (n = 2083)	P
Implantation rate	749/2323(32.24)	1367/3817 (35.81)	0.004
Biochemical pregnancy rate	645/1268(50.87)	1156/2083 (55.50)	0.009
Clinical pregnancy rate	596/1268(47.00)	1059/2083 (50.84)	0.031
Miscarriage rate	55/596 (9.23)	94/1059 (8.88)	0.810
1st trimester	48/596(8.05)	82/1059 (7.74)	0.822
2st trimester	7/596(1.17)	12/1059(1.13)	0.94
Ectopic pregnancy rate	14/596(2.35)	30/1059(2.83)	0.557
Multiple pregnancy rate	144/596(24.16)	304/1059(28.71)	0.046
Ongoing pregnant rate	527/1268(41.56)	935/2083(44.89)	0.06
Live birth rate	506/1268(39.91)	900/2083(43.21)	0.06

Obstruction group vs. embolization group

Data are presented as mean ± SD for continuous variables and n (%) for dichotomous variables

P < 0.05 indicated a significant statistical difference

There were no marked differences in the live birth rate between two groups, even after adjustment of potentially confounding factors. After adjustment for some confounding factors, however, as compared to the embryos transferred at blastocysts stage (day 5), the live birth rate significantly decreased after transfer with cleavage stage (day 3) (aOR 0.73; 95% CI 0.567–0.935). Women with thicker endometrium on the day of transfer (aOR 1.07; 95% CI 1.022–1.124) and more embryos transferred (aOR 1.42; 95% CI 1.033–1.945) had significantly increased live-birth rate. Women with more embryos transferred at blastocysts stage (day 5), thicker endometrium on the day of transfer (aOR 1.01; 95% CI 1.006–1.106) and more embryos transferred (aOR 1.41; 95% CI 1.033–1.918) had significantly increased rate of clinical pregnancy. The covariates adjusted in the multiple Logistic model for live birth and clinical pregnancy rates are listed in Table 4.

**Table 4:** Results of multiple regression analysis for live birth rates

	aOR (LB)	95%CI (LB)	P (LB)	aOR (CP)	95%CI (CP)	P (CP)
Tubal obstruction	Reference					
Tubal embolization	0.89	0.692–1.145	0.366	0.84	0.657–1.076	0.169
Age	0.96	0.928–1.020	0.170	0.96	0.919–1.003	0.117
BMI	0.99	0.962–1.034	0.938	1.01	0.961–1.038	0.764
Infertility duration	0.96	0.913–1.020	0.170	0.957	0.910–1.006	0.238
Gravidity	0.98	0.764–1.243	0.836	1.01	0.976–1.285	0.927
Parity	1.42	0.880–2.287	0.151	1.47	0.912–2.385	0.114
Main cause of infertility						
Tubal factor	Reference					
PCOS	0.56	0.123–2.563	0.457	0.72	0.157–3.267	0.667
Endometriosis	0.93	0.175–4.990	0.937	1.17	0.220–6.266	0.851
Male factor	0.46	0.092–2.297	0.345	0.61	0.123–3.001	0.540
Diminish ovary reserve	0.54	0.117–2.523	0.436	0.72	0.155–3.340	0.675
Number of previous FET	0.94	0.745–1.172	0.559	0.93	0.774–1.161	0.519
Fertilization method	0.98	0.808–1.200	0.876	1.00	0.823–1.215	0.999
Number of oocytes retrieved	1.09	0.935–1.258	0.283	1.10	0.952–1.275	0.195
FET endometrial preparation	1.08	0.941–1.244	0.270	1.07	0.935–1.227	0.321
Endometrium thickness on the day of transfer (mm)	1.07	1.022–1.124	0.004	1.01	1.006–1.106	0.027
Number of embryos transferred	1.42	1.033–1.945	0.031	1.41	1.033–1.918	0.03
Embryo developmental stage at transfer (D3)	0.73	0.567–0.935	0.013	0.71	0.547–0.912	0.008

Analyses were performed after adjustment for age, infertility duration, gravidity, parity, main cause of infertility, number of OPU prior to FET, year of treatment, and number of embryos transferred, and embryo developmental stage at transfer

*AOR* adjusted odd ratio; *FET* frozen-thawed embryo transfer; *OR* odd ratio; *PCOS* polycystic ovary syndrome. Implantation rate was defined as the number of gestational sacs visualized on ultrasound examination divided by the number of embryos transferred. Ongoing pregnancy rate was defined as a viable pregnancy that progressed beyond 12 weeks of gestational divided by the number of embryos transferred for each group. Live birth rate was defined as the number of live births divided by the number of embryos transfer for each group

The neonatal outcomes after the first treatment cycle following the freeze-all strategy among singletons are presented in Table 5. The rate of neonatal death (0.59% [embolization group] vs 0.11% [control group]), stillbirth (0.20% vs 0.22%), gestational age, birth weight were comparable between embolization group and control group among singletons and twins (Table 6).

**Table 5:** Neonatal outcomes after different managements of hydrosalpinx among singletons

Characteristics	Embolization group (n = 1268)	Obstruction group (n = 2083)	P
Stillbirth	1/506(0.20)	3/900(0.22)	0.647
Neonatal death	3/506(0.59)	1/900(0.11)	0.104
Weeks of gestation at birth (weeks)			0.036
Normal, 37 +	359/424 (84.67)	619/788(78.55)	
Preterm, 32–37	57/424 (13.44)	150/788(19.04)	
Early preterm, < 32	8/424 (1.89)	19/788(2.41)	
Birth weight (kg)			0.849
Normal birth weight, 2.5–4.0	325/366(88.80)	692/778(88.95)	
Very low birth weight, < 1.5	2/366 (0.55)	8/778(1.03)	
Low birth weight, 1.5–2.5	12/366 (3.28)	28/778(3.60)	
High birth weight, 4.0–4.5	25/366 (6.83)	43/778(5.53)	
Very high birth weight, > 4.5	2/366 (0.55)	7/778(8.90)	

Obstruction group vs. embolization group

Data are presented as mean ± SD for continuous variables and n (%) for dichotomous variables

P < 0.05 indicated a significant statistical difference

**Table 6:** Neonatal outcomes after different managements of hydrosalpinx among twins

Characteristics	Embolization group (n = 1268)	Obstruction group (n = 2083)	P
Birth weight (kg)			0.686
Normal birth weight, 2.5–4.0	153/252 (60.71)	45/75 (60%)	
Very low birth weight, < 1.5	11/618 (1.78)	5/75 (6.67)	
Low birth weight, 1.5–2.5	91/252 (36.11)	25/75 (34.67)	
High birth weight, 4.0–4.5	0/252 (4.05)	0/75 (0)	
Very high birth weight, > 4.5	0/252 (0)	0/75 (0)	

Data are expressed as n (%)

*Adjusted for age, infertility type, causes of infertility, and type of cycle (IVF/intracytoplasmic sperm injection)

P < 0.05 indicated a significant statistical difference

## Discussion

In this large retrospective study, the impacts of interventional embolization of hydrosalpinx on the pregnancy outcomes were investigated in women undergoing FET with high-quality embryo transfer. Our results indicated that, based on a freeze-all policy, interventional embolization of hydrosalpinx could achieve the same live birth rate as hydrosalpinx-free obstruction with less risk, less pain and less cost. However, significant reduction was found in the implantation rate (35.81% [obstruction group] vs 32.24% [embolization group]; P = 0.004). Although the endometrial receptivity of hydrosalpinx patients was different from that of hydrosalpinx-free obstruction patients, the implantation rate was lower and the live birth rate was not affected in the embolization group. This suggests that embolization of hydrosalpinx is a preferred clinical treatment for hydrosalpinx.

Current treatments for hydrosalpinx mainly include ultrasound-guided hydrosalpinx aspiration, fimbria salpingostomy, proximal tubal ligation, and tubectomy before IVF-ET. Simple hydrosalpinx aspiration usually fails to improve the clinical pregnancy rate, implantation rate and parturition rate of IVF-ET, but may cause hydrosalpinx relapse [16, 17]. Salpingostomy often causes a high rate of hydrosalpinx recurrence in a short period and high incidences of abortion and ectopic gestation [13]. Tubectomy and proximal tubal ligation can significantly improve the clinical pregnancy rate and reduce the ectopic pregnancy rate. However, they are easy to cause damage to the mesovarium artery arches, which affects the ovarian blood supply and reduces the ovarian reserve function and superovulation response [8]. In the interventional embolization, microcoil is placed between the interstitial section and the isthmus of fallopian tube. The mechanism of action may be as follows: (1) mechanically complete partition of tubular lumina and (2) alkaline phosphatase released from the mildly mechanical necrotic tissues can benefit lymphocytic aggregation and proliferation of vascular tissues, which increases the embolization of tubular lumina [14]. A mechanical method in the interventional embolization of fallopian tube has no effect on the ovarian function.

Hydrosalpinx has an adverse effect on the outcomes after IVF-ET [18, 19]. To date, there is no consensus regarding the best surgical management for hydrosalpinx before IVF-ET. In the present study, the live-birth outcomes after FET were compared between hydrosalpinx patients treated with interventional embolization and hydrosalpinx-free obstruction patients. Compared with interventional embolization group, significantly higher implantation rate and clinical pregnancy rate were observed in the hydrosalpinx-free obstruction group. The flow of hydrosalpinx liquid may affect embryo implantation by mechanically flushing the uterine cavity. In addition, there are micro-organisms, toxic debris, lymphocytes, cytokines, prostaglandins, and other agents in the hydrosalpinx fluid. These may also transfer directly to the uterine cavity, exerting deleterious effects on the embryos or endometrium [20–22]. Based on our results, the significantly higher implantation rate and clinical pregnancy rate may be related to more embryos transferred at blastocysts stage (day 5) in the control group (17.71% vs 9.22%, P < 0.05). The endometrial receptivity of patients with interventional embolization was not different from that of patients with hydrosalpinx-free tubal infertility.

Moreover, there were no significant differences in the rates of ongoing pregnancy and live birth in patients with interventional embolization for hydrosalpinx. Both groups had similar live birth rate. After adjustment for confounding factors, compared to the embryos transferred in the blastocysts stage (day 5), the live birth rate significantly decreased after transfer with cleavage stage (day 3). Women with thicker endometrium on the day of transfer and more embryos transferred had a significantly higher live birth rate. For the baseline characteristics, significant difference was noted in cause of infertility. After adjustment for confounding factors, compared with the tubal factor, there were no impact on the live birth and clinical outcomes in case of high-quality embryo transferring. For patients with polycystic ovary syndrome (PCOS) or premature ovarian failure, the protocol and dose of drugs in egg retrieval cycle may be completely different. However, in the FET cycle, we usually use the letrozole ovulation protocol to prepare the endometrium in patients with PCOS. In a study of Di Paola et al. they calculated the follicle-stimulating hormone (FSH) starting dose according to the nomogram [23]. Their results indicated nomogram in intrauterine insemination (IUI) cycles could lead to a more tailored FSH starting dose and improve cost-effectiveness, especially in the expected hyper-response patients, which may reduce hyper-response and cancellation rate, and increase optimal follicle retrieval. The menstrual cycle is short and the basic FSH is high for patients with premature ovarian failure, and therefore hormone replacement treatment is preferred. Seckin et al. concluded that low-dose gonadotropin treatment was advised for women with PCOS, and there was no significant difference in pregnancy rates of non-obese patients with PCOS having IUI whether rFSH was started at 37.5 or 75 international units (IU) [24]. In addition, there is evidence showing that obese women require significantly larger amounts of gonadotropins to achieve similar IVF success rates as normal weighting women [25]. The pregnancy outcomes are not affected in case of high-quality embryos transferred [12]. It has been reported that interventional embolization can prevent tubal fluid flowing toward the cavity and improve the pregnancy outcomes of IVF [26]. Interventional embolization of hydrosalpinx can achieve the same live birth rate as hydrosalpinx-free obstruction with less risk, less pain and less cost.

In the salpingectomy, chronically infected tissues are removed, which limits the risk of subsequent abscess formation or torsion, as well as improves the access to the ovaries during the egg retrieval in the IVF [27]. However, laparoscopy is an invasive procedure, which may be infeasible in the presence of dense adhesions. In addition, laparoscopy has the risk of damaging major organs or vessels, especially in patients with previous abdominal/pelvic surgery, severe endometriosis or inflammatory bowel disease. In the study, the interventional embolization combined with ultrasound-guided hydrosalpinx puncture has been a routine treatment of hydrosalpinx in our center. If hydrosalpinx is identified on ultrasonography before transplantation, hydrosalpinx will be routinely punctured under ultrasound examination 2 days before transplantation. If the hydrosalpinx recurs on the day of transplantation, it is punctured again. If the hydrosalpinx is mild, hydrosalpinx is punctured on the day of transplantation, but uterus puncturing should be avoided, in order to not induce uterine contraction, or it will affect transplantation. Currently, the success rate of FET with the guidance of transvaginal or transabdominal ultrasound is controversial [28, 29]. In a recent study, Cozzolino et al. found a moderate quality of evidence supporting the beneficial effects of transabdominal guidance during embryo transfer compared with conventional clinical touch in clinical pregnancy and ongoing or live birth rates; quality of evidence supporting the equivalence of transvaginal vs transabdominal approach in clinical pregnancy and ongoing or live birth rates to be low [28]. Larue et al. found transvaginal ultrasound guidance of the transfer significantly increased the percentage of pregnancies per transfer compared with transfers performed under transabdominal ultrasound guidance; transvaginal ultrasound facilitated the performance of difficult transfers and in particular achieved outcomes in the situations that are not significantly different from those of easy transfers [29]. In the present study, FET was conducted under the guidance of transabdominal ultrasound. This procedure is minimally invasive, and easy and quick to perform. In addition, the retrograde flow of the hydrosalpingeal fluid, a factor related to the perturbed uterine environment, is eliminated by this procedure, and the integrity of ovarian blood supply is maintained. However, the interventional embolization is associated with ongoing pelvic pain secondary to the pressure and the diseased tube, and has risks of adnexal torsion and subsequent need for adnexal surgery [30]. Moreover, it has reported the clip migration and chronic pelvic pain post procedure [27]. In this study, there were no pelvic inflammation attack, accessory torsion and accessory abscess during pregnancy after interventional embolization. Therefore, the long-term safety during pregnancy after interventional embolization should be further investigated in studies with large sample size. The embolization may cause aseptic inflammation around the microcoil to block the accumulation of hydrosalpinx. In the present study, the ectopic pregnancy rate in the interventional embolization group was similar to that in the hydrosalpinx-free group, indicating that interventional embolization of hydrosalpinx before IVF-ET can reduce the ectopic pregnancy rate. Salpingectomy may also increase the risk of interstitial pregnancy (IP) (pregnancy within the fallopian tube that is located in the uterine wall and connects with the remainder of the tube to the endometrial cavity) in patients when the transection of the fallopian tube is close to the cornua. In a study of Wang et al. among 43 patients with IP, 71% underwent bilateral salpingectomy before IVF [31]. A corneal suture placed on laparoscopic salpingectomy has been shown to reduce the risk of IP, increasing the intrauterine pregnancy rate, ongoing pregnancy rate and live birth rate as compared to those without placement of a corneal suture [32]. In this study, ectopic pregnancy was noted in 14 patients and cornual pregnancy in 2 patients with tubal embolization. The ectopic pregnancy was found at the affected side in 1 patient. Three patients with ligation of oviduct developed ectopic pregnancy at the fallopian tube. There was no IP in the patients receiving interventional embolization for hydrosalpinx. The causes of ectopic pregnancy in hydrosalpinx patients are still unclear and may include the placement of microcoil, drop of microcoil, and hysteroscopy. Interventional embolization is considered as an alternative management, especially in patients with distorted pelvic anatomy or severe pelvic adhesions. At present, torsion during pregnancy and pelvic inflammation were not found in the patients of present study. One patient had ectopic microcoil. Polyethylene terephthalate fibers, which run through the inner coil of the Essure® device, may cause tissue reaction, resulting in tubal occlusion when the coil has been inserted. A systematic review of 11 studies (115 women who received Essure®) has shown successful placement in 96.5% of patients and the tubal occlusion was observed in 98.1% of patients [9]. With subsequent IVF, a reasonable pregnancy rate of 38.6% and live birth rate of 27.9% were achieved, and investigators concluded that Essure® is effective for the management of hydrosalpinx in women before IVF, when operative treatment is limited by pelvic adhesions. Compared with Essure®, microcoil is used for interventional embolization in HX, with a failure rate of < 5%. The microcoil used in the present study was made of titanium alloy. It can induce the oviducal inflammation, resulting in the oviducal obstruction, which avoids the flow of fluid in the hydrosalpinx into the uterus and therefore increases the success rate. However, a two-center randomized controlled trial (RCT) (involving 85 women) shows that, before IVF/ICSI, hysteroscopic tubal occlusion is inferior to laparoscopic salpingectomy; the ongoing pregnancy rate per patient following proximal interventional embolization with intratubal devices is 26.2% as compared to 55.8% following laparoscopic salpingectomy [33]. Furthermore, a systematic review involving more than 3000 patients shows that management of hydrosalpinx by hysteroscopic placement of Essure® devices before IVF produces lowers clinical pregnancy rate and live birth rate than those by laparoscopic salpingectomy and laparoscopic proximal tubal occlusion [26]. These results are inconsistent with our findings. Therefore, we recommend interventional embolization of hydrosalpinx as a treatment for hydrosalpinx. The adverse effects of hydrosalpinx on the uterine milieu during the implantation window have been well described. Current guidance supports the use of interventional embolization for tubal occlusion in women with hydrosalpinx before IVF or ICSI, as there is higher rate of successful treatment and the pregnancy rate is improved. Individualized care, based on woman's risk profile and preferences, is required to help make clinical decision, considering the full range of treatment options.

Our study shows that interventional embolization followed by IVF-ET can effectively improve the adverse effects of hydrosalpinx on the embryos and significantly increase the clinical pregnancy rate of IVF-ET while reducing the ectopic pregnancy rate. This is consistent with clinical findings after IVF-ET with ultrasound-guided aspiration of hydrosalpinx during the controlled ovarian hyperstimulation. The effects of interventional embolization on the IVF-ET before and after controlled ovarian hyperstimulation remain to be further studied. In our center, the interventional embolization is generally performed after controlled ovarian hyperstimulation.

There were several limitations in the present study. It was a retrospective study, the database was screened with strict inclusion criteria, and only patients receiving first embryo transfer cycle in our center were included for analysis. Additionally, the vast majority of patients in the present study received transfer of two cleavage-stage embryos, rather than single blastocyst transfer. Chinese legislation limited the proportion of blastocyst transfer cycles within 7%, aiming to control the male birth. Thus, the transfer of two cleavage-stage embryos remains a priority in Chinese IVF centers [34, 35]. In addition, the number of patients with the drop of microcoil, which needs to be removed by hysteroscopy, was not determined in the present study.

The present study had several strengths. To the best of our knowledge, this is a study with the largest sample size that evaluates the impact of hydrosalpinx on the reproductive outcomes. A number of potential confounders that may bias the results are controlled in the present study. Furthermore, all the data are from a single center, and thus the practice consistency can be assured. Except for the type of culture medium, all other IVF procedures and laboratory conditions remain unchanged during the study period.

## Conclusion

In summary, our study indicates that interventional embolization of hydrosalpinx is one of the preferred clinical treatments for hydrosalpinx. More studies are warranted to investigate the short- and long-term effects of interventional embolization on the pregnancy outcomes and neonates, which may provide more information about the management of hydrosalpinx.

## Data Availability

The data used to support the findings of this study are available from the corresponding author upon request*.*

## Abbreviations

AC: Artificial cycle
CIs: Confidence intervals
FET: Frozen-thawed embryo transfer
HSG: Hystero-salpingography
ICSI: Intracytoplasmic sperm injection
IVF-ET: In vitro fertilization and embryo transfer
LBW: Low birth weight
OPU: Ovum pick-up
PTB: Premature term baby
